# A Schema for Digitized Surface Swab Site Metadata in Open-Source DNA Sequence Databases

**DOI:** 10.1128/msystems.01284-22

**Published:** 2023-02-27

**Authors:** Jingzhang Feng, Devin Daeschel, Damion Dooley, Emma Griffiths, Marc Allard, Ruth Timme, Yi Chen, Abigail B. Snyder

**Affiliations:** a Department of Food Science, Cornell University, Ithaca, New York, USA; b Centre for Infectious Disease Genomics and One Health, Faculty of Health Sciences, Simon Fraser University, Burnaby, British Columbia, Canada; c Division of Microbiology, Office of Regulatory Science, Center for Food Safety and Applied Nutrition, U.S. Food and Drug Administration, College Park, Maryland, USA; University of California San Diego

**Keywords:** genomic surveillance, epidemiology, informatics, foodborne pathogen

## Abstract

Large, open-source DNA sequence databases have been generated, in part, through the collection of microbial pathogens by swabbing surfaces in built environments. Analyzing these data in aggregate through public health surveillance requires digitization of the complex, domain-specific metadata that are associated with the swab site locations. However, the swab site location information is currently collected in a single, free-text, “isolation source”, field-promoting generation of poorly detailed descriptions with various word order, granularity, and linguistic errors, making automation difficult and reducing machine-actionability. We assessed 1,498 free-text swab site descriptions that were generated during routine foodborne pathogen surveillance. The lexicon of free-text metadata was evaluated to determine the informational facets and the quantity of unique terms used by data collectors. Open Biological Ontologies (OBO) Foundry libraries were used to develop hierarchical vocabularies that are connected with logical relationships to describe swab site locations. 5 informational facets that were described by 338 unique terms were identified via content analysis. Term hierarchy facets were developed, as were statements (called axioms) about how the entities within these five domains are related. The schema developed through this study has been integrated into a publicly available pathogen metadata standard, facilitating ongoing surveillance and investigations. The One Health Enteric Package was available at NCBI BioSample, beginning in 2022. The collective use of metadata standards increases the interoperability of DNA sequence databases and enables large-scale approaches to data sharing and artificial intelligence as well as big-data solutions to food safety.

**IMPORTANCE** The regular analysis of whole-genome sequence data in collections such as NCBI’s Pathogen Detection Database is used by many public health organizations to detect outbreaks of infectious disease. However, isolate metadata in these databases are often incomplete and of poor quality. These complex, raw metadata must often be reorganized and manually formatted for use in aggregate analyses. These processes are inefficient and time-consuming, increasing the interpretative labor needed by public health groups to extract actionable information. The future use of open genomic epidemiology networks will be supported through the development of an internationally applicable vocabulary system with which swab site locations can be described.

## INTRODUCTION

**FIG 1:**
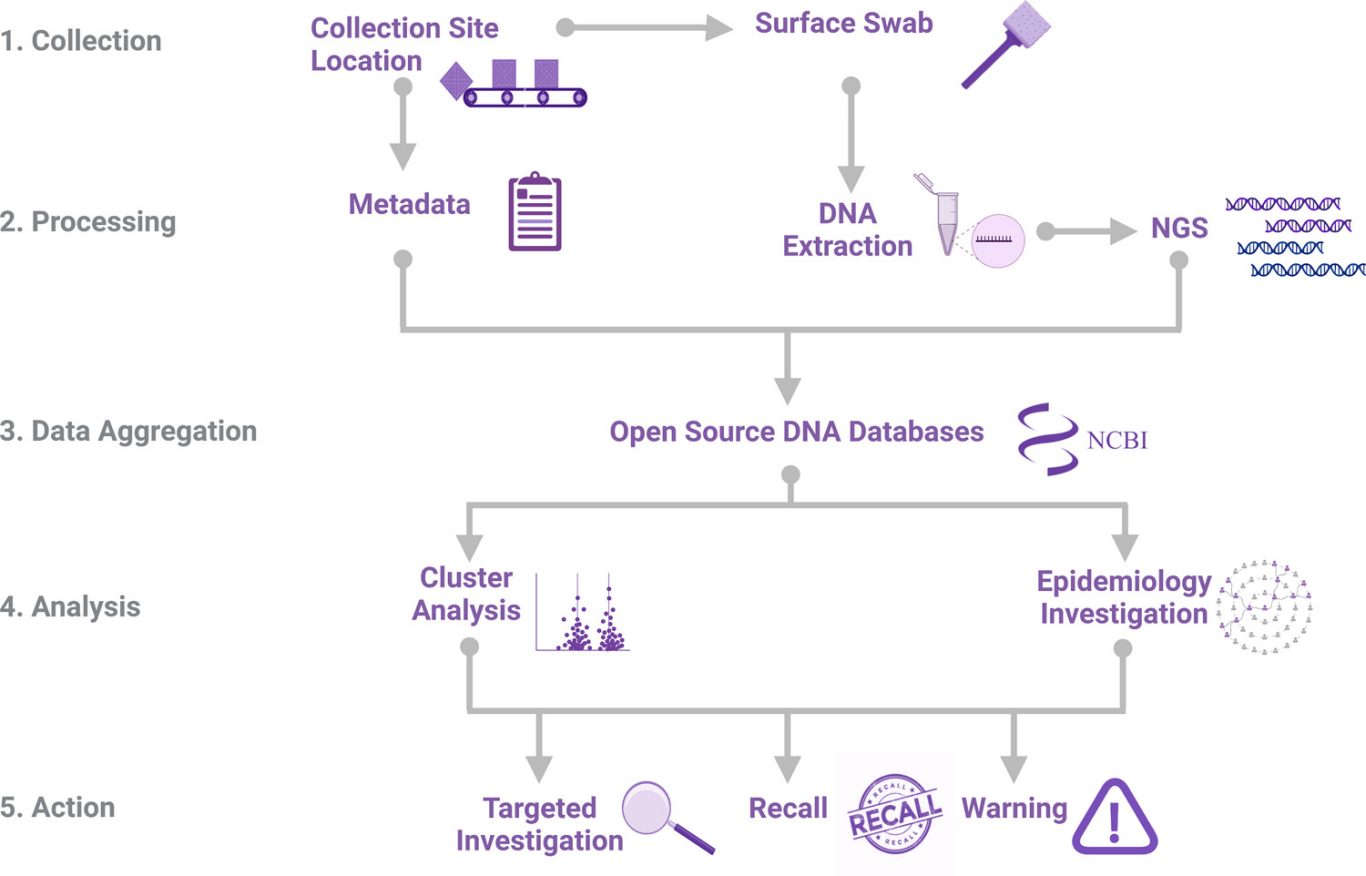
An overview of infectious disease surveillance and outbreak investigations that use WGS and associated source metadata. WGS, whole-genome sequencing.

The modern surveillance of foodborne pathogens is reliant on large, open-source DNA sequence databases. Examples of laboratory networks that contribute to these databases through ongoing surveillance programs include GenomeTrakr and PulseNet. Regular analyses of whole-genome sequence (WGS) data in collections, such as NCBI’s Pathogen Detection Database, are used by many public health organizations to detect outbreaks of infectious diseases (1). In these analyses, the identification of pathogens from the environment that cluster with pathogens from sickened individuals informs epidemiological investigations (2). Subsequently, the metadata that are associated with environmental pathogens directly supports the identification of point sources (Fig. 1). Regulatory environmental monitoring activities involve the collection of surface swabs within built environments, and this is followed by an evaluation of the infectious pathogens that are collected on the swabs (3, 4). The metadata that describe these swab site locations provides the context for pathogen isolation sources and must provide sufficient detail in order to be actionable (2, 5, 6). In addition to contextualizing data from individual samples, environmental monitoring metadata enables large collections of genomic data to be shared and integrated through digital networks (7). The realization of such open genomic epidemiology networks has been hindered by the absence of an internationally applicable vocabulary system with which swab site locations can be described.

Despite its importance, isolate metadata in large, open-source DNA sequence databases are often incomplete and of poor quality. Metadata include general attributes (date, geographic location) as well as domain-specific attributes (swab site location, collection method) (2, 8, 9). Descriptions of swab site locations within built environments are particularly challenging to standardize due to the complexity and variation of the necessary information. Consequently, they are currently reported by individual data collectors as unstructured, free-text responses. These complex, raw metadata must often be reorganized and manually formatted into a uniform pattern in order for them to be usable in aggregate analyses. Primary data collectors must sometimes be contacted during outbreak investigations for additional information. These processes are inefficient and time-consuming, and they increase the interpretative labor needed by public health groups to extract actionable information to address urgent public health needs (2). In contrast, improved metadata standards support the FAIR principles of data management in that they enable the increased Findability, Accessibility, Interoperability, and Reusability of large, open-source DNA sequence data (9).

The goal of this study was to use a collection of unstructured, free-text swab site location descriptions to inform the development of a schema with which to structure and standardize swab site location metadata. Such a system will be integrated within broader minimum metadata standards for built environments and will be used by data collectors within public health groups (7, 10, 11). To accomplish this goal, the Open Biological Ontologies (OBO) Foundry Principles (12) were used to develop a schema that defined (1) informational facets, (3) ontologized terms, and (4) statements (called axioms) about how entities within informational facets were related in order to better structure and standardize descriptions of environmental monitoring swab site locations. We then applied this schema in a use case of Listeria monocytogenes from different food production environments. Collectively, this analysis allowed us to identify gaps in existing ontologies, such as a lack of terms for industrial equipment, and to create resources that were appropriate for our use case and are applicable for reuse in the analyses of other types of data sets so as to better harmonize and integrate public health food safety research.

## RESULTS AND DISCUSSION

### Lexicon of unstructured, free-text metadata.

Here, we present an assessment of the lexicon typically used by data collectors in unstructured, free-text descriptions of swab site locations. By anatomizing these responses (breaking down the descriptions into discrete concepts), we identified common language structures as well as recurrent issues. These findings informed the development of the standardized schema that is described below. Within the free-text responses, the frequent use of synonyms and presence of occasional typos complicated machine-readability in aggregate analyses. We have used a conveyor belt as an illustrative example in this article because it is a common and complex structure that is often associated with the harborage of L. monocytogenes through surface swabbing. In free-text responses, data collectors variously referred to this structure as a “conveyor”, “conveyor system”, “processing line”, “conveyor belt”, “belt”, or simply by the brand name of the equipment, and a common misspelling was “conveyer”. Moreover, swab site descriptions were not single terms (e.g., “conveyor”), but rather short descriptive statements, such as “leg of the conveyor with rusted hole”, which contained several different facets of information. This complexity contributes to the challenge of the machine interpretation of swab site location descriptions. In addition to the potential complexity, free-text statements were also often incomplete or imprecise. For example, in the description “condensation coming down next to conveyor”, it is unclear what surface structure was swabbed, beyond that it had condensation on it, which complicates even human interpretation.

**FIG 2:**
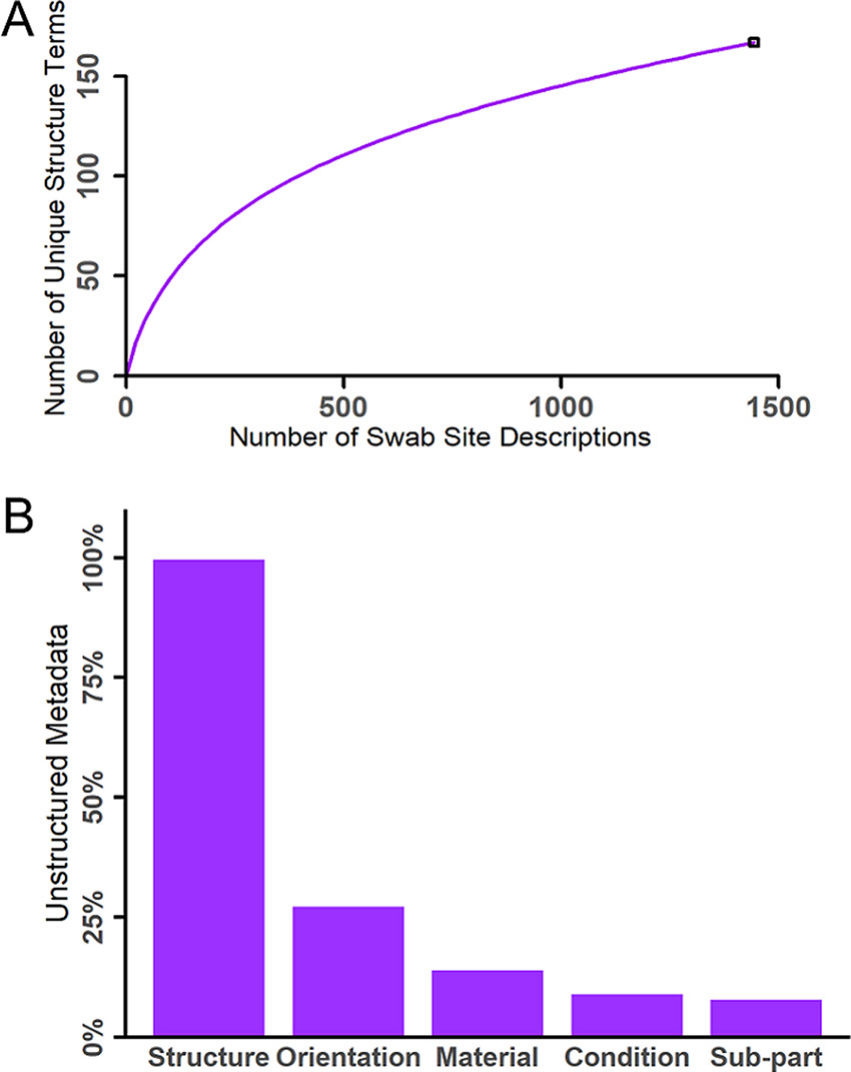
An analysis of the free-text swab site location descriptions revealed (A) a high level of term diversity and (B) a low degree of completeness across information facets.

Through the assessment of the term diversity used in free-text descriptions, we identified 338 unique terms used within 1,498 swab site descriptions. The majority of these terms (*n* = 253) were used to describe the structure and the subpart of the structure that was swabbed. A rarefaction curve illustrating the increase in the number of unique structure terms as a function of the number of the total swab site descriptions (Fig. 2A) revealed a high level of term diversity being used to describe surface structures (13, 14). Significant term diversity represents a challenge in the standardization of metadata format and signals an ongoing need for management and curation (15–17). As a consequence of this finding, the adoption of controlled terms, such as in a community-supported ontology, may be warranted, as an ontology provides clear definitions and promotes consistent application across data collectors (18, 19).

While most of the unique terms used in free-text responses referred to the structure, several other informational facets that described important aspects of the surface environment were commonly included. In total, we identified five unique informational facets: (i) the structure being swabbed, (ii) the subpart of the structure, (iii) the material from which the swab surface was composed, (iv) the condition of the surface, and (v) the orientation of the swab site location on the structure. As an example, “underside of the cracked plastic belt of the conveyor” includes all five information facets. An assessment of how consistently each of these informational facets were addressed across unstructured, free-text responses revealed that while the structure was defined in nearly all of the metadata (99.6%), the remaining informational facets were addressed far less frequently (Fig. 2B). This suggests that the completeness of swab site descriptions could be improved by prompting data collectors for specific informational facets. In contrast, metadata that are dumped into a single “isolation source” field triggers the collection of short, less detailed descriptions in which word order, granularity, spelling, and the use of synonyms or abbreviations vary. In epidemiological investigations, the analysis and interpretation of the swab site information impacts the speed and scope of the public health response (2). Incomplete or uninterpretable metadata increases the difficulty of identifying the origins of infectious pathogens, which complicates root cause analyses (20).

### Ontology reuse and axiom construction.

**FIG 3:**
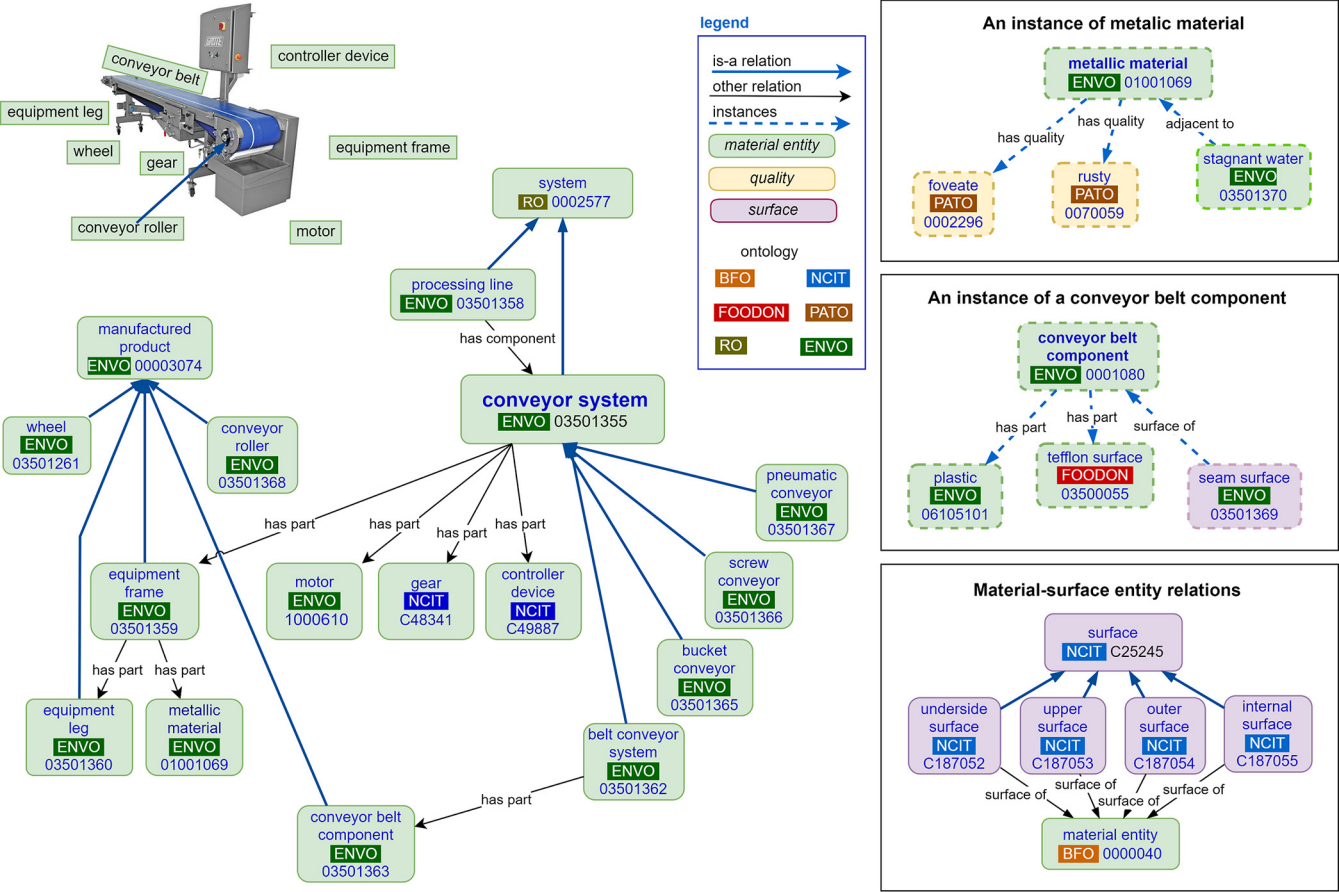
A semantic model illustrating the logical relationships among the unique terms representing the different informational facets that were used to describe the swab site locations on a conveyor belt.

Ontologies are one solution to the challenges that are associated with free-text responses. By defining the terms in a hierarchical structure, ontologies standardize the definition for each term. For instance, the term “conveyor system” falls under the parent category “system” (Fig. 3). This hierarchy defines how a conveyor belt is related to other types of manufacturing equipment, which is important for swab site descriptions that contain different levels of granularity. Comparisons among metadata that vary in granularity are otherwise difficult without substantial text mining, and they are currently limited by agency-specific classification schemes, which are essentially flat lists. Because ontological terms are connected through logical reasoning, the definitions for these terms are more explicit and encode knowledge within a specific domain (12, 19).

Most of the terms we identified in the free-text responses were transformed from the Environment Ontology (ENVO) or the Phenotype and Trait Ontology (PATO). ENVO contains specialized terms for industrialized equipment and manufacturing applications, and this supports the informational facets that are related to the structure or the subpart of the structure being swabbed (16). PATO contains terms describing the characteristics of material things, and this supports the informational facets that are related to the type of surface material and its condition. Both are mature, community-supported ontologies. Ontologies enable community consensus about classification schemes and the meanings of descriptors. The use of mature, community-supported ontologies enables the ongoing management and curation of term collection as new descriptors are proposed by individual users or by groups of users, as needed. For example, the FoodOn consortia is composed of academic, regulatory, and industry partners from many countries and product-domains. This group handles food product and processing terms as well as conceptual contributions from the public via tickets submitted to the FoodOn GitHub. A similar process exists for ENVO, which also offers a point of contact for non-ontologists to directly submit term proposals via email.

Historically, metadata have not been documented using ontologies, and terms used in free-text metadata are less nuanced. OBO Foundry principles encourage the reuse of terms from other ontologies wherever applicable (21, 22). This prevents redundant or conflicting efforts and maximizes the benefits of community coordinated efforts in the maintenance of centralized registries. The use of third-party ontologies allows users to access and retrieve terms from specialist domain ontologies. Also, when needed, new terms can be proposed. For example, we proposed several new terms to ENVO, such as “equipment leg” (ENVO: 03501360) and “conveyor roller” (ENVO: 03501368), and to PATO, including “rusted” (PATO: 0070059). These terms had not been previously included, but they were relevant to our use case. Term proposal is a necessary and continuous effort in the adoption of an ontology approach. For example, we found gaps in ENVO due to a lack of terms describing manufacturing equipment. Similarly, we found that terms in PATO centered on biological systems and did not capture all of the nuances of the characteristics describing abiotic surfaces. Our term proposal focused on addressing these gaps, and this will be an ongoing necessity for dedicated user groups.

The adoption of ontologies standardizes the use of terms by data collectors, thereby enforcing accurate referencing. Terms are linked to OBO ontology identification numbers and object properties. This avoids the use of ambiguous free-text responses. Controlled definitions promote consistent application, in contrast to the examples of misspellings, synonyms, and colloquialisms that are often observed in unstructured, free-text responses (23). Moreover, these definitions within term hierarchies are managed through cross-cultural and expert consensus in use case domains, ensuring broader understanding and agreement (22, 24). We developed a semantic model (Fig. 3) to illustrate the conceptual relationship among the informational facets that are commonly used to describe swab site locations. Individual terms are connected with axioms, which are statements about how entities within a domain are related (25–27). These interconnections are held by the Relation Ontology (RO) and are represented by the arrows shown in Fig. 3 (28). For example, the “has part” RO statement establishes the “conveyor roller” as a subpart of the larger “conveyor system”, whereas the “has quality” statement allows references to be made to material condition features through various terms, including “pitted”, “rusted”, or having “standing water”, as relevant. It is notable in this example that some of the “has quality” characteristics can be described as “instances” and are therefore represented by dashed lines within this visualization (Fig. 3). The use of instances implies that these characteristics, such as “rusted”, apply to some but not all metallic materials. Ultimately, this framing supports the machine-readability of metadata by assigning ontology identification numbers that can be recognized digitally and support the computer recognition of semantic relationships (29).

### Application in the “smarter” era of food safety.

**FIG 4:**
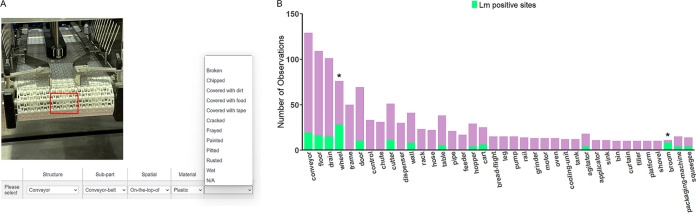
Standardized swab site location metadata supports public health initiatives. (A) Digital tools, such as applications with selectable drop-down menus, can assist data collectors with real-time data capture. (B) Machine-readable metadata support the rapid assessments of sampling efforts and pathogen detection. The green shading corresponds to the number of swab sites that were positive for Listeria monocytogenes. An asterisk (*) indicates a structure in which L. monocytogenes was significantly overrepresented.

The schema described here has been implemented in the One Health Enteric Package, which is an expanded and standardized suite of metadata for the genomic surveillance of enteric pathogens (30). This package was developed by a U.S. interagency working group, namely, GenFS (31), with the goal of expanding and standardizing the metadata that are collected for sample types that span the One Health continuum: humans, animals, and environments, including built environments (32). Additional efforts to develop minimum metadata schemes to contextualize surface (drag) swabs from natural environments, among other sources, are being conducted by this group. Ontology-based packages, such as these, make comparisons across other ontology-based schemes that are developed by other agencies (with overlapping but slightly different scopes) more mappable and easier to compare. For example, the U.S. interagency One Health Enteric Package may be more readily compared with the One Health AMR standard that is being developed by a joint-agency Canadian initiative (33). Complete and consistent metadata enhance the efficiency of epidemiological investigations, as evidenced by other groups who have successfully applied ontology-based approaches, including National Center for Biotechnology Information (NCBI) and European Molecular Biology Laboratory-European Bioinformatics Institute (EMBL-EBI) (22, 34). However, to our knowledge, this is the first schema that has been developed to capture machine-readable descriptions of swab site locations in built environments, building on the MixS minimum metadata standard for built environments (7). In application, data collectors can draw upon this schema to standardize vocabulary usage in their domain of interest. However, this also requires buy-in from a broad base of data collectors, who may need to expend more effort in the generation of standardized metadata, compared to the level of effort that is currently needed to generate free-text responses. While this upfront effort ultimately benefits public health by expediting investigations and reducing interpretative labor (2), buy-in from data collectors can also be enhanced by reducing the barriers to adopting best practices. For example, application-based tools that increase the ease of metadata collection will increase buy-in (Fig. 4A). Training, technical support, and a shared conceptual understanding of the value of these upfront efforts are also important to adoption. These efforts are more generally a part of the U.S. Food and Drug Administration’s framework that is outlined in the “New Era of Smarter Food Safety Blueprint” and targets the tech-enabled traceability and adoption of smarter tools and approaches for prevention and outbreak responses (31). In that vein, the broader applicability of frameworks that standardize metadata will increase automation and machine-actionability.

Digitized metadata also support more fundamental initiatives that are driven by machine learning, artificial intelligence, and big data approaches to food safety, beyond simply facilitating outbreak investigations. The Internet of things (IoT) requires machine-readable metadata and offers interconnection across people (e.g., public health agencies, industry, consumers) and machines (e.g., automated notification systems, open-source DNA sequence repositories, high-throughput bioinformatic pipelines) (22). As a brief example, digitized metadata can enable quantitative evaluation of surveillance efforts themselves. Decisions regarding which locations to sample are largely made by individuals, but machine-readable metadata allows for the evaluation of those selections in aggregate. From our data set, we could easily identify which structures were most often selected for sampling, once the metadata had been standardized and digitized (Fig. 4B). We could then assess the L. monocytogenes positivity rate among the most common swab site structures. Within this data set, wheels (28 out of 128) and brooms (8 out of 11) had significantly higher proportions of samples that were positive for L. monocytogenes (*P* < 0.05), compared to other commonly selected locations. Although this example alone does not reflect a sufficient and balanced sample on which to base policy, it illustrates how this type of analysis can drive improvement in targeting swab site locations as agencies iterate on their previous findings to identify high-risk locations with increasing specificity. In contrast, analyzing these questions from free-text metadata would be prohibitively time-consuming, as NCBI’s Pathogen Detection database currently contains over 50,000 L. monocytogenes entries. As the rate of WGS data collection has increased dramatically over the last decade, it is crucial to adopt practices that ensure complete and high-quality metadata as quickly as possible for current and future database management (35).

### Conclusion.

Outbreak investigations rely on the contextual information that describes the sources of environmental pathogens to track the origins of outbreaks of infectious diseases. These metadata should be interpretable by humans and machines, and they should provide sufficient detail for analyzing the sources of pathogens. Based on our assessment of previously generated free-text responses that describe swab site locations within built environments, we have identified common issues and have proposed a schema for prospective, standardized metadata collection. Although textual assessments, such as these, are both time-intensive and labor-intensive, this study models a possible process for the development of subsequent metadata schema. The direct application of such a framework in future data collection consequently mitigates many challenges that are associated with a reliance on text mining, such as the restrictions on some texts due to copyright legislation and the avoidance of the necessary reformatting and preprocessing of documents, prior to analysis. This schema capitalizes on the framework from existing ontologies and formalizes data capture in five major informational facets. The resources developed here are also compatible with FoodOn (22) and GenEpio (36), which facilitates the integration of genomic epidemiological data with food product data. The One Health Enteric Metadata Package, hosted at NCBI BioSample, includes this framework for samples that were collected during facility inspections, in effect, implementing the schema that is recommended here for the national and international genomic surveillance of foodborne pathogens. International alignment on contextualizing metadata from pathogen surveillance is increasingly important in a food system with global value chains.

## MATERIALS AND METHODS

### Collection and evaluation of unstructured, free-text metadata.

We studied 1,498 swab site descriptions that were generated during routine food safety surveillance and investigation activities by the U.S. Food and Drug Administration (FDA). All information specific to the facilities was anonymized. These records were from a total of 9 facilities and were taken on 22 different collection dates that were selected via convenience sampling. The sites included one dairy, four produce, one seafood, and one mixed facility. All of the text from the swab site metadata was extracted using the text mining tool (tm) v0.7-8 in R version 4.1.2 (37). The free-text responses were anatomized by categorizing the components of each description into different data facets. In addition to extracting word-frequency counts, content analysis was used to analyze the themes and patterns of the free-text responses, based on explicit rules (38). Briefly, emergent coding was performed by two researchers who independently reviewed the free-text metadata and identified a set of features that formed an initial checklist. The researchers then used the consolidated checklist to independently code features in the free-text metadata. An example of how different concepts were formalized as informational fields is illustrated in Fig. S1. After independently coding features, the agreement between the researchers was >95%. In cases of disagreement, the coding was discussed, and a consensus on the assignment was identified.

10.1128/msystems.01284-22.1 FIG S1 The metadata from unstructured swab site descriptions were categorized based on the following scheme. (A) An example showing how an unstructured swab site description was anatomized, based on this scheme. (B) Descriptions and examples for the metadata categories in the swab site descriptions. Out of these categories, structure, subpart, orientation, material, and condition were identified as the five minimum required categories for metadata describing swab sites. Location terms were not included in the analysis. (C) Illustration of how free-text terms were placed into hierarchical structures within ontologies. Download FIG S1, DOCX file, 0.09 MB. Copyright © 2023 Feng et al. 2023 Feng et al. https://creativecommons.org/licenses/by/4.0/ This content is distributed under the terms of the Creative Commons Attribution 4.0 International license.

### Proposing new terms to OBO.

Each unique free-text term was queried in Ontobee, a type of the Ontology Lookup Service (OLS) (39). Terms that were not found in Ontobee were proposed as new terms within existing Open Biological Ontologies (OBO). Proposed new terms were each linked to an existing OBO term, which served as the parent class representing the broader hierarchical category in which the new term would be categorized. For example, the term “rusted” was linked to “texture” in the Phenotype and Trait Ontology (PATO). In addition, the textual definition of the proposing term was generated, following the Aristotelian format (40). The parent classes and textual definitions of terms were documented in a ROBOT (12) template, as shown in Table S1. A summary of the information that must be documented within ROBOT templates to propose new OBO terms can be found in Table S2. In total, we requested 21 term additions to 3 ontologies. 15 of these term requests were sent to ENVO, 1 request was made to PATO, and the remaining 5 requests were to the NCI Thesaurus (NCIT). All of the requested terms were fulfilled in their corresponding ontologies. The ROBOT files were submitted to the relevant ontologies through their GitHub pages. Following the submission, ontology curators initiated a quality check (QC) procedure to ensure that the semantics of the proposed terms were compatible with those of the existing terms. Terms passing the QC check were assigned an ID number and were integrated within the ontologies. The larger framework connecting these terms, which is described in detail within Results, utilized logical relationships from the Relation Ontology (RO), as summarized in Table S3.

10.1128/msystems.01284-22.2 TABLE S1 A ROBOT template that lists the terms that were uploaded to the Environment Ontology (ENVO). Download Table S1, XLSX file, 0.01 MB. Copyright © 2023 Feng et al. 2023 Feng et al. https://creativecommons.org/licenses/by/4.0/ This content is distributed under the terms of the Creative Commons Attribution 4.0 International license.

10.1128/msystems.01284-22.3 TABLE S2 The information required for proposing terms to existing ontologies, based on OBO principles. The example here is for the proposed entity “conveyor”. Download Table S2, DOCX file, 0.02 MB. Copyright © 2023 Feng et al. 2023 Feng et al. https://creativecommons.org/licenses/by/4.0/ This content is distributed under the terms of the Creative Commons Attribution 4.0 International license.

10.1128/msystems.01284-22.4 TABLE S3 Terms in the Relation Ontology (RO) that describe logical relations between entities that are relevant to swab site locations. Download Table S3, DOCX file, 0.01 MB. Copyright © 2023 Feng et al. 2023 Feng et al. https://creativecommons.org/licenses/by/4.0/ This content is distributed under the terms of the Creative Commons Attribution 4.0 International license.

### Statistical analysis.

A rarefaction curve was generated using the vegan package v2.5-7 with a subset of 20 unique descriptions to evaluate the diversity of terms collected from the free-text swab site metadata (41). The swab site locations within our collection where L. monocytogenes was detected were analyzed as an example use case for our schema. This data set is publicly available in Table S2, and the L. monocytogenes genomic data are available on NCBI through the identified SRR numbers. A chi-square test of association with a Bonferroni correction was performed in R v4.1.2 to identify structure locations with statistically significantly higher incidences of L. monocytogenes. The Bonferroni correction was applied to adjust for the multiple comparisons among 20 structures.

10.1128/msystems.01284-22.5 TABLE S4 The SRR number for each *Listeria* WGS in NCBI. Download Table S4, XLSX file, 0.02 MB. Copyright © 2023 Feng et al. 2023 Feng et al. https://creativecommons.org/licenses/by/4.0/ This content is distributed under the terms of the Creative Commons Attribution 4.0 International license.

## References

[1] Amezquita A, Barretto C, Winkler A, Baert L, Jagadeesan B, Lewenthal D-A, Klijin A. 2020. The benefits and barriers of whole-genome sequencing for pathogen source tracking: a food industry perspective. Food Saf Mag.

[2] Pettengill JB, Beal J, Balkey M, Allard M, Rand H, Timme R. 2021. Interpretative labor and the bane of nonstandardized metadata in public health surveillance and food safety. Clin Infect Dis 73:1537–1539. doi:10.1093/cid/ciab615. 34240118

[3] De Filippis F, Valentino V, Alvarez-Ordóñez A, Cotter PD, Ercolini D. 2021. Environmental microbiome mapping as a strategy to improve quality and safety in the food industry. Curr Opin Food Sci 38:168–176. doi:10.1016/j.cofs.2020.11.012.

[4] Jones SL, Ricke SC, Keith Roper D, Gibson KE. 2020. Swabbing the surface: critical factors in environmental monitoring and a path towards standardization and improvement. Crit Rev Food Sci Nutr 60:225–243. doi:10.1080/10408398.2018.1521369. 30421977

[5] Linkert M, Rueden CT, Allan C, Burel J-M, Moore W, Patterson A, Loranger B, Moore J, Neves C, MacDonald D, Tarkowska A, Sticco C, Hill E, Rossner M, Eliceiri KW, Swedlow JR. 2010. Metadata matters: access to image data in the real world. J Cell Biol 189:777–782. doi:10.1083/jcb.201004104. 20513764 PMC2878938

[6] Afolayan AO, Bernal JF, Gayeta JM, Masim ML, Shamanna V, Abrudan M, Abudahab K, Argimón S, Carlos CC, Sia S, Ravikumar KL, Okeke IN, Donado-Godoy P, Aanensen DM, Underwood A, Harste H, Kekre M, Muddyman D, Taylor B, Wheeler N, David S, Arevalo A, Fernanda VM, Osma CE, Nagaraj G, Govindan V, Prabhu A, Sravani D, Shincy MR, Rose S, Ravishankar KN, Oaikhena AO, Ajiboye JJ, Ewomazino OE, Lagrada ML, Macaranas PKV, Olorosa AM, Herrera EM, Molloy A, Stelling J, Vegvari C, NIHR Global Health Research Unit on Genomic Surveillance of Antimicrobial Resistance. 2021. Overcoming data bottlenecks in genomic pathogen surveillance. Clin Infect Dis 73:S267–S274. doi:10.1093/cid/ciab785. 34850839 PMC8634317

[7] Yilmaz P, Kottmann R, Field D, Knight R, Cole JR, Amaral-Zettler L, Gilbert JA, Karsch-Mizrachi I, Johnston A, Cochrane G, Vaughan R, Hunter C, Park J, Morrison N, Rocca-Serra P, Sterk P, Arumugam M, Bailey M, Baumgartner L, Birren BW, Blaser MJ, Bonazzi V, Booth T, Bork P, Bushman FD, Buttigieg PL, Chain PSG, Charlson E, Costello EK, Huot-Creasy H, Dawyndt P, DeSantis T, Fierer N, Fuhrman JA, Gallery RE, Gevers D, Gibbs RA, Gil IS, Gonzalez A, Gordon JI, Guralnick R, Hankeln W, Highlander S, Hugenholtz P, Jansson J, Kau AL, Kelley ST, Kennedy J, Knights D, Koren O, et al. 2011. Minimum information about a marker gene sequence (MIMARKS) and minimum information about any (x) sequence (MIxS) specifications. Nat Biotechnol 29:415–420. doi:10.1038/nbt.1823. 21552244 PMC3367316

[8] Singh G, Bharathi S, Chervenak A, Deelman E, Kesselman C, Manohar M, Patil S, Pearlman L. 2003. A metadata catalog service for data intensive applications, p 33. *In* Proceedings of the 2003 ACM/IEEE conference on Supercomputing - SC ’03. ACM Press, Phoenix, AZ.

[9] Wilkinson MD, Dumontier M, Aalbersberg IJJ, Appleton G, Axton M, Baak A, Blomberg N, Boiten J-W, da Silva Santos LB, Bourne PE, Bouwman J, Brookes AJ, Clark T, Crosas M, Dillo I, Dumon O, Edmunds S, Evelo CT, Finkers R, Gonzalez-Beltran A, Gray AJG, Groth P, Goble C, Grethe JS, Heringa J, 't Hoen PAC, Hooft R, Kuhn T, Kok R, Kok J, Lusher SJ, Martone ME, Mons A, Packer AL, Persson B, Rocca-Serra P, Roos M, van Schaik R, Sansone S-A, Schultes E, Sengstag T, Slater T, Strawn G, Swertz MA, Thompson M, van der Lei J, van Mulligen E, Velterop J, Waagmeester A, Wittenburg P, et al. 2016. The FAIR Guiding Principles for scientific data management and stewardship. Sci Data 3:160018. doi:10.1038/sdata.2016.18. 26978244 PMC4792175

[10] Perez-Riverol Y, European Bioinformatics Community for Mass Spectrometry. 2020. Toward a sample metadata standard in public proteomics repositories. J Proteome Res 19:3906–3909. doi:10.1021/acs.jproteome.0c00376. 32786688 PMC7116434

[11] Glass EM, Dribinsky Y, Yilmaz P, Levin H, Van Pelt R, Wendel D, Wilke A, Eisen JA, Huse S, Shipanova A, Sogin M, Stajich J, Knight R, Meyer F, Schriml LM. 2014. MIxS-BE: a MIxS extension defining a minimum information standard for sequence data from the built environment. ISME J 8:1–3. doi:10.1038/ismej.2013.176. 24152717 PMC3869023

[12] Jackson RC, Balhoff JP, Douglass E, Harris NL, Mungall CJ, Overton JA. 2019. ROBOT: a tool for automating ontology workflows. BMC Bioinformatics 20:407. doi:10.1186/s12859-019-3002-3. 31357927 PMC6664714

[13] Hughes JB, Hellmann JJ. 2005. The application of rarefaction techniques to molecular inventories of microbial diversity, p 292–308. *In* Methods in Enzymology. Elsevier, Alpharetta, GA. 10.1016/S0076-6879(05)97017-1 16260298

[14] Koellner T, Hersperger AM, Wohlgemuth T. 2004. Rarefaction method for assessing plant species diversity on a regional scale. Ecography 27:532–544. doi:10.1111/j.0906-7590.2004.03832.x.

[15] Andrés-Hernández L, Baten A, Azman Halimi R, Walls R, King GJ. 2020. Knowledge representation and data sharing to unlock crop variation for nutritional food security. Crop Sci 60:516–529. doi:10.1002/csc2.20092.

[16] Buttigieg P, Morrison N, Smith B, Mungall CJ, Lewis SE, the ENVO Consortium. 2013. The environment ontology: contextualising biological and biomedical entities. J Biomed Semantics 4:43. doi:10.1186/2041-1480-4-43. 24330602 PMC3904460

[17] Eftimov T, Ispirova G, Potočnik D, Ogrinc N, Koroušić Seljak B. 2019. ISO-FOOD ontology: a formal representation of the knowledge within the domain of isotopes for food science. Food Chem 277:382–390. doi:10.1016/j.foodchem.2018.10.118. 30502161

[18] Schuurman N, Leszczynski A. 2006. Ontology-based metadata. Trans GIS 10:709–726. doi:10.1111/j.1467-9671.2006.01024.x.

[19] Lumsden J, Hall H, Cruickshank P. 2011. Ontology definition and construction, and epistemological adequacy for systems interoperability: a practitioner analysis. J Inf Sci 37:246–253. doi:10.1177/0165551511401804.

[20] Black A, MacCannell DR, Sibley TR, Bedford T. 2020. Ten recommendations for supporting open pathogen genomic analysis in public health. Nat Med 26:832–841. doi:10.1038/s41591-020-0935-z. 32528156 PMC7363500

[21] Jackson R, Matentzoglu N, Overton JA, Vita R, Balhoff JP, Buttigieg PL, Carbon S, Courtot M, Diehl AD, Dooley DM, Duncan WD, Harris NL, Haendel MA, Lewis SE, Natale DA, Osumi-Sutherland D, Ruttenberg A, Schriml LM, Smith BCJS, Jr, Vasilevsky NA, Walls RL, Zheng J, Mungall CJ, Peters B. 2021. OBO Foundry in 2021: operationalizing open data principles to evaluate ontologies. Database 2021:1–9. doi:10.1093/database/baab069. PMC8546234 34697637

[22] Dooley DM, Griffiths EJ, Gosal GS, Buttigieg PL, Hoehndorf R, Lange MC, Schriml LM, Brinkman FSL, Hsiao WWL. 2018. FoodOn: a harmonized food ontology to increase global food traceability, quality control and data integration. NPJ Sci Food 2:23. doi:10.1038/s41538-018-0032-6. 31304272 PMC6550238

[23] Chan L, Vasilevsky N, Thessen A, McMurry J, Haendel M. 2021. The landscape of nutri-informatics: a review of current resources and challenges for integrative nutrition research. Database 2021:baab003. doi:10.1093/database/baab003. 33494105 PMC7833928

[24] Vitali F, Lombardo R, Rivero D, Mattivi F, Franceschi P, Bordoni A, Trimigno A, Capozzi F, Felici G, Taglino F, Miglietta F, De Cock N, Lachat C, De Baets B, De Tré G, Pinart M, Nimptsch K, Pischon T, Bouwman J, Cavalieri D, ENPADASI consortium. 2018. ONS: an ontology for a standardized description of interventions and observational studies in nutrition. Genes Nutr 13:12. doi:10.1186/s12263-018-0601-y. 29736190 PMC5928560

[25] Taye M. 2010. Understanding semantic web and ontologies: theory and applications. J Comput 2:182–192.

[26] Uschold M, Gruninger M. 1996. Ontologies: principles, methods and applications. Knowl Eng Rev 11:93–136. doi:10.1017/S0269888900007797.

[27] Villa F, Athanasiadis IN, Rizzoli AE. 2009. Modelling with knowledge: a review of emerging semantic approaches to environmental modelling. Environ Model Softw 24:577–587. doi:10.1016/j.envsoft.2008.09.009.

[28] Smith B, Ceusters W, Klagges B, Köhler J, Kumar A, Lomax J, Mungall C, Neuhaus F, Rector AL, Rosse C. 2005. Relations in biomedical ontologies. Genome Biol 6:R46. doi:10.1186/gb-2005-6-5-r46. 15892874 PMC1175958

[29] Griffiths E, Dooley D, Graham M, Van Domselaar G, Brinkman FSL, Hsiao WWL. 2017. context is everything: harmonization of critical food microbiology descriptors and metadata for improved food safety and surveillance. Front Microbiol 8:1068. doi:10.3389/fmicb.2017.01068. 28694792 PMC5483436

[30] Timme RE, Wolfgang WJ, Balkey M, Venkata SLG, Randolph R, Allard M, Strain E. 2020. Optimizing open data to support one health: best practices to ensure interoperability of genomic data from bacterial pathogens. One Health Outlook 2:20. doi:10.1186/s42522-020-00026-3. 33103064 PMC7568946

[31] U.S. Food & Drug Administration. 2022. New era of smarter food safety - FDA’s blueprint for the future. https://www.fda.gov/food/new-era-smarter-food-safety.

[32] Stevens EL, Carleton HA, Beal J, Tillman GE, Lindsey RL, Lauer AC, Pightling A, Jarvis KG, Ottesen A, Ramachandran P, Hintz L, Katz LS, Folster JP, Whichard JM, Trees E, Timme RE, McDERMOTT P, Wolpert B, Bazaco M, Zhao S, Lindley S, Bruce BB, Griffin PM, Brown E, Allard M, Tallent S, Irvin K, Hoffmann M, Wise M, Tauxe R, Gerner-Smidt P, Simmons M, Kissler B, Defibaugh-Chavez S, Klimke W, Agarwala R, Lindsay J, Cook K, Austerman SR, Goldman D, McGARRY S, Hale KR, Dessai U, Musser SM, Braden C. 2022. Use of whole genome sequencing by the Federal Interagency Collaboration for Genomics for Food and Feed Safety in the United States. J Food Prot 85:755–772. doi:10.4315/JFP-21-437. 35259246

[33] Griffiths E, Rhiannon C, Sehar A. 2022. GRDI_AMR_One_Health. Github. https://github.com/cidgoh/GRDI_AMR_One_Health.

[34] McMahon C, Denaxas S. 2016. A novel framework for assessing metadata quality in epidemiological and public health research settings. AMIA Jt Summits Transl Sci Proc 2016:199–208. 27570670 PMC5001774

[35] Kush R, Goldman M. 2014. Fostering responsible data sharing through standards. N Engl J Med 370:2163–2165. doi:10.1056/NEJMp1401444. 24897080

[36] Dooley D, Griffiths E, Gosal G, Brinkman F, Hsiao W. 2017. The Genomic Epidemiology Ontology and GEEM Ontology Reusability Platform. Academia 6.

[37] Feinerer I, Hornik K, Meyer D. 2008. Text mining infrastructure in *R*. J Stat Softw 25:1–54.

[38] Stemler S. 2001. An overview of content analysis. Pract Assess Res Eval 7:1–6.

[39] Jupp S, Burdett T, Malone J, Leroy C, Pearce M, Parkinson H. 2015. A new ontology lookup service at EMBL-EBI. Proceedings of SWAT4LS International Conference 2015. Cambridge, United Kingdom.

[40] OBO Foundry. 2022. Principle: textual definitions (principle 6). OBO Foundry. https://obofoundry.org/principles/fp-006-textual-definitions.html.

[41] Okasanen J, Blanchet FG, Friendly M, Kindt R, Legendre P, McGlinn D, Minchin PR, O’Hara RB, Simpson GL, Solymos P, Henry M, Stevens H, Szoecs E, Wagner H. 2018. Community Ecology Package. R package version 2.6–2.

