# Supplementary figures and images for: A Schema for Digitized Surface Swab Site Metadata in Open-Source DNA Sequence Databases
Source: mSystems. 2023 Feb 27;8(2):e01284-22. doi: 10.1128/msystems.01284-22 (PMC10134794; doi:10.1128/msystems.01284-22)

**A**

**
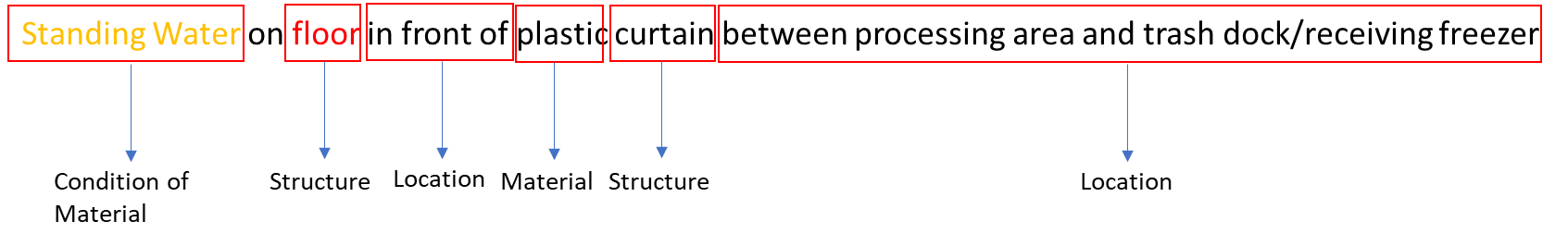
**

**B**

**
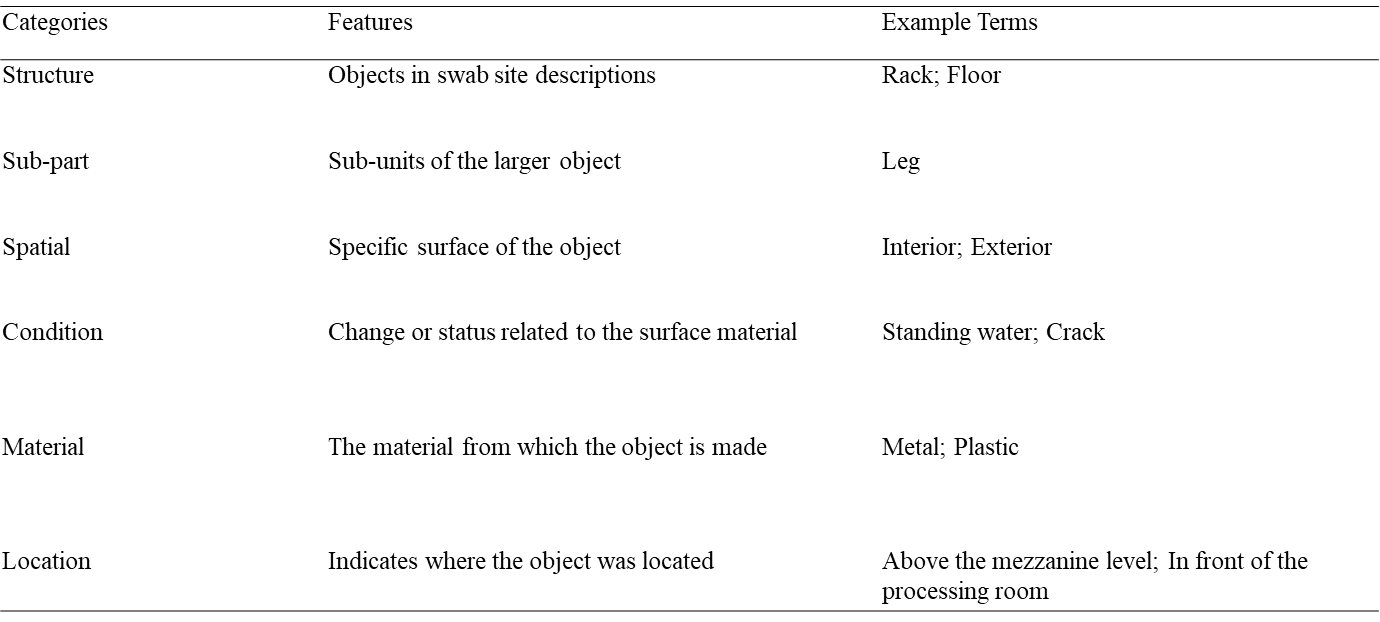
**

**C**

**
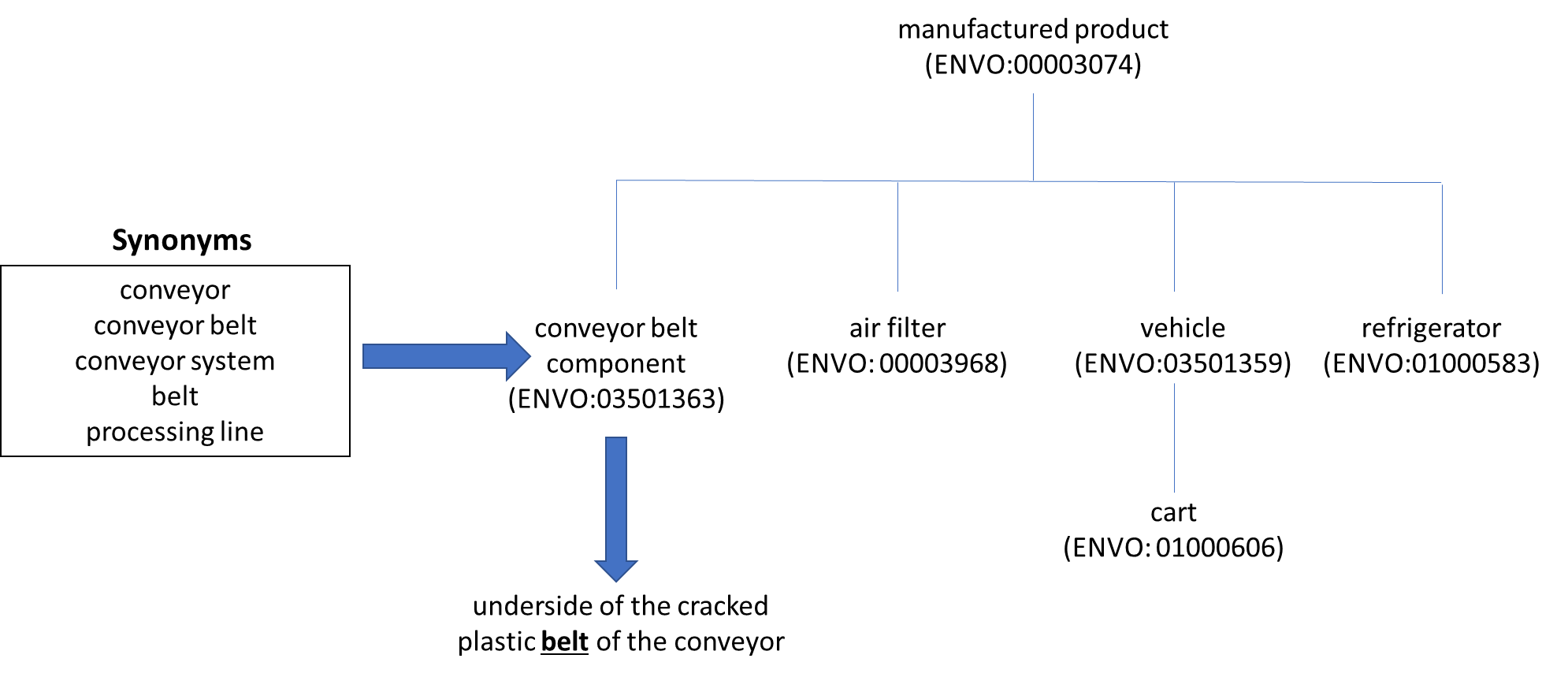
**

**Figure S1.**

Supplement: FIG S1 [file msystems.01284-22-s0003.docx]
