# Supplementary material for: A Schema for Digitized Surface Swab Site Metadata in Open-Source DNA Sequence Databases
Source: mSystems. 2023 Feb 27;8(2):e01284-22. doi: 10.1128/msystems.01284-22 (PMC10134794; doi:10.1128/msystems.01284-22)
Supplement: TABLE S2 [file msystems.01284-22-s0004.docx]

**Table S2**

| **Components** | **Description** | **Example** | **ROBOT label** |
| --- | --- | --- | --- |
| Ontology ID | An assigned ID number to represent the identity of the proposing term | ENVO:03501356 | ID |
| Label | The term that is proposing to the ontology | Conveyor system | A label |
| Parent class | The parent class of the proposing term | System | SC % |
| Definition | The definition of the ontology label in the form of [A X which has/is Y] | A system which is composed of one or more machines that can continuously transport material from one location to at least one other location. | AL IAO:0000115@en |
| Definition Cross Reference | Referenced source for the definition of the object | <http://vocab.getty.edu/page/aat/300024559> | >AI oboInOwl:hasDbXref SPLIT=\| |
| Comment | Additional information of the proposing term |  | AL rdfs:comment@en |
| Comment cross reference | Source of the additional information |  | >AI oboInOwl:hasDbXref SPLIT=\| |
| Editors note | Additional notes for ontology editors to better engineer to proposing term |  | AL IAO:0000116@en |
| Exact synonym | Other terms with the exact same meaning of the proposing term | conveyor equipment | AL oboInOwl:hasExactSynonym@en SPLIT=\| |
| Broad synonym | Synonyms with similar meaning as the proposing term but could also refer to other objects | conveyor | AL oboInOwl:hasBroadSynonym@en SPLIT=\| |
| Narrow synonym | Synonyms that refer to something more specific than the proposing term | food processing conveyor | AL oboInOwl:hasNarrowSynonym@en SPLIT=\| |
| Related synonym | Synonyms that could be narrow and broad sysnonyms |  | AL oboInOwl:hasRelatedSynonym@en SPLIT=\| |
| In subset | The community in which the proposing term is pertinent to | food safety | A oboInOwl:inSubset SPLIT=\| |
| Cross reference | Source providing the information of the proposing term |  | AI oboInOwl:hasDbXref SPLIT=\| |
| Subclass axiom | Other logical axioms helping to define the proposing term |  | SC % |
| Creation date | Date where the term was uploaded to ontologies | 2021-12-09T00:00:00Z | AT oboInOwl:creation_date^^xsd:dateTime |
