# Supplementary material for: A Schema for Digitized Surface Swab Site Metadata in Open-Source DNA Sequence Databases
Source: mSystems. 2023 Feb 27;8(2):e01284-22. doi: 10.1128/msystems.01284-22 (PMC10134794; doi:10.1128/msystems.01284-22)
Supplement: TABLE S3 [file msystems.01284-22-s0005.docx]

**Table S3**

| **Logical Relationships** | **Description** |
| --- | --- |
| is_a | Represents the relationship in which an entity is the upper-class hierarchy of another object |
| has_component | Represents the relationship in which an entity is a sub-unit of another entity |
| material_surface_of | Represents the relationship in which an entity is a specific surface of another entity |
| has_part | Represents the relationship in which an entity is the material of another entity |
| has_quality | Represents the relationship in which an entity is the temporary state of a surface material |
